# The Combination of SMAD4 Expression and Histological Grade of Dysplasia Is a Better Predictor for the Malignant Transformation of Oral Leukoplakia

**DOI:** 10.1371/journal.pone.0066794

**Published:** 2013-06-24

**Authors:** Rong-Hui Xia, Xiao-Meng Song, Xiao-Jing Wang, Jiang Li, Li Mao

**Affiliations:** 1 Department of Oral Pathology, 9th People's Hospital, Shanghai Jiao Tong University, School of Medicine, Shanghai Key Laboratory of Stomatology, Shanghai, China; 2 Department of Oncology and Diagnostic Sciences, Dental School, University of Maryland, Baltimore, Maryland, United States of America; 3 Department of Oral Maxillofacial–Head and Neck Oncology, Ninth People's Hospital, Shanghai Jiao Tong University, School of Medicine, Shanghai Key Laboratory of Stomatology, Shanghai, China; 4 Department of Pathology, University of Colorado School of Medicine, Aurora, Colorado, United States of America; Johns Hopkins University, United States of America

## Abstract

Oral leukoplakia (OL) is the most common premalignancy in the oral cavity and can progress to oral squamous cell carcinoma (OSCC). SMAD4 is a tumor suppressor implicated in multiple cancer types including OSCC. To assess the role of SMAD4 in oral leukoplakia malignant transformation, the authors investigated SMAD4 expression patterns in OL and OSCC using a highly specific antibody and correlated the patterns with the risk of malignant transformation oral leukoplakia. Immunohistochemistry and a quantitative imaging system were used to measure SMAD4 expression in OL from 88 OL patients, including 22 who later went through malignant transformation, and their OSCC counterpart. Forty-three (48.9%) of the 88 OL patients had strong SMAD4 expression. SMAD4 expression had no significant correlation with patients' clinicopathological parameters. Interestingly, 17 (39.5%) of the 43 OL lesions with strong SMAD4 expression went through malignant transformation whereas only 5 (11.1%) of the 45 OL lesions with weak SMAD4 expression did so (p = 0.002). The SMAD4 expression in OL was much higher than that in their OSCC counterpart. Kaplan-Meier analysis revealed that the combination of SMAD4 expression and histological grade of dysplasia (p = 0.007) is a better predictor for the malignant transformation of oral leukoplakia. In the multivariate analysis, both SMAD4 expression and grade of dysplasia were identified as independent factors for OL malignant transformation risk (p = 0.013 and 0.021, respectively). It was concluded that high SMAD4 expression may be indicative of an early carcinogenic process in OL and serve as an independent biomarker in assessing malignant transformation risk in patients with OL, and the combination of SMAD4 expression and histological grade of dysplasia is a better predictor for the malignant transformation of oral leukoplakia.

## Introduction

Oral squamous cell carcinoma (OSCC), which comprises approximately half of head and neck cancer, is the most common subtype of head and neck carcinoma[1], [2]. The 5-year survival rate of patients with OSCC remains almost unchanged despite various treatment improvements in the last three decades[3]. Oral leukoplakia (OL) is described as a white patch and cannot be characterized as any other disease clinically or histologically[4], [5]. OL represents the most common oral precancerous condition with 17% to 35% of the lesion going through malignant transformation[6], [7].

Currently, histopathological assessment for the grade of epithelial dysplasia is the most important method to determine malignant potential of patients with OL[8]. However, grading epithelial dysplasia is subjective, and the predictive value of dysplasia for OL malignant transformation is poor. Due to the high morbidity and mortality of advanced stage OSCC, there is an urgent need to develop biomarkers independent of histopathological assessment for the prediction.

Mammalian SMADs were discovered in 1996 and named after their non-mammalian homolog, Sma and Mad[9]. SMAD proteins could be phosphorylated and activated by transmembrane serine-threonine receptor kinases in response to TGF-beta stimulation [10]. SMAD family was classified as receptor SMAD (SMAD 1/2/3/5/8), common SMAD (SMAD4) and inhibitory SMAD (SMAD6/7). The product of SMAD4 gene forms homomeric complexes and heteromeric complexes with other activated SMAD proteins, such as SMAD2 and SMAD3, which then accumulate in the nucleus and regulate the transcription of target genes. Previous studies demonstrated that SMAD4 was considered as a tumor suppressor and was frequently mutated or homozygously deleted in pancreatic cancer and colorectal cancer [11], [12], [13]. Loss expression of SMAD4 was associated with poor clinical outcomes in patients with pancreatic, colon, and brain cancers[14], [15], [16]. Although mutation and homozygous deletion of SMAD4 were rare in head and neck squamous cell carcinoma (HNSCC)[17], knockout SMAD4 could lead to spontaneous oral squamous cell carcinoma development in an animal model[18], suggesting the tumor suppressor role of SMAD4 in oral tumorigenesis.

In this study, we intended to determine the expression pattern of SMAD4 in OL and its potential clinical implications as a biomarker for OL malignant transformation. We examined SMAD4 expression pattern in OL lesions from 88 patients, who had a mean follow-up time of 76.18 months. The SMAD4 expression pattern and other clinicopathological parameters were analyzed to determine their value as biomarkers for predicting the risk of OL malignant transformation.

## Results

### Patient clinicopathological characteristics

Detailed clinicopathological characteristics and follow-up information for each patient are presented in Table S1. Of the 88 patients with OL, 37 (42%) were male and 51 (58%) were female with age ranging from 27 to 85 years old (mean 56 years). Among the 88 OL lesions, 57 (65%) were located at tongue and 31 (35%) were located at other anatomic area in oral cavity. During the average 76.18 months follow-up period, 22 of 88 (25%) OL lesions went through malignant transformation after the initial diagnosis. The 88 patients with OL in the current cohort were grouped as malignant transformed (n = 22) and untransformed(n = 66) cases. The grade of dysplasia showed a slight difference between these two groups as the p-value was 0.047, while age, gender, lesion site, smoking history and alcohol intake showed no significant difference. The detailed clinicopathological characteristics of the patients with OL were summarized in Table 1.

**Table 1:** Clinicopathologic characteristics and SMAD4 expression of patients with OL.

		Oral leukoplakia		
	No. of Patient	MT(%)	UT(%)	p value
All patients	88	22 (25.0)	66 (75.0)	
Age (years)				
≤55	47	11 (23.4)	36 (76.6)	0.711
>55	41	11 (26.8)	30 (73.2)	
Sex				
Male	37	6 (16.2)	31 (83.8)	0.105
Female	51	16 (31.4)	35 (68.6)	
Grade of dysplasia				
High	22	9 (40.9)	13 (59.1)	**0.047**
Low-moderate	66	13 (19.7)	53 (80.3)	
Lesion site				
Nontongue	31	9 (29.0)	22 (71.0)	0.519
Tongue	57	13 (22.8)	44 (77.2)	
Smoking				
Never	64	19 (29.7)	45 (70.3)	0.214
Past and present	16	2 (12.5)	14 (87.5)	
Unknown	8			
Alcohol intake				
Never	64	18 (28.1)	46 (71.9)	0.538
Past and present	17	3 (17.6)	14 (82.4)	
Unknown	7			
SMAD4 expression				
Strong	43	17 (39.5)	26 (60.5)	**0.002**
Weak	45	5 (11.1)	40 (88.9)	

Abbreviations: MT, malignant-transformed oral leukoplakia; UT: untransformed oral leukoplakia.

Bold indicates values that are statistically significant (p<0.05).

### SMAD4 expression in OL and OSCC samples

**Figure 1:**
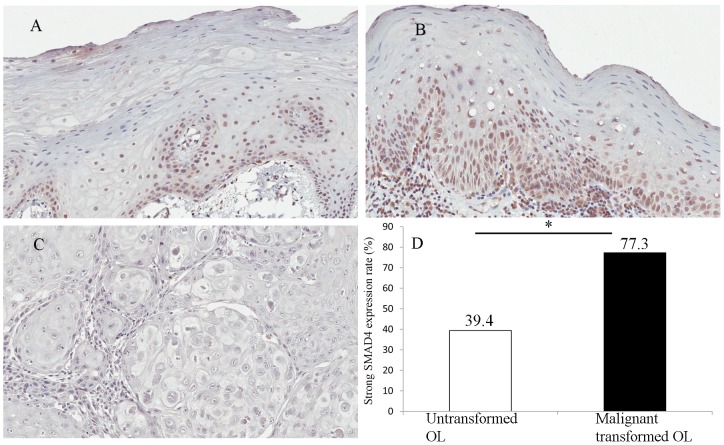
Expression pattern of SMAD4 in OL and OSCC samples. A, case 31: weak expression of SMAD4 in OL lesion which did not go through malignant transformation (x20). B, case 59:strong expression of SMAD4 in OL lesion which later went through malignant transformation (x20). C, OSCC samples which derived from OL lesion: weak expression of SMAD4 in OSCC samples. D, The frequency of strong SMAD4 expression is 39.4% in untransformed OL and 77.3% in malignant transformed OL. * indicated that p = 0.002.

SMAD4 expression was measured in the 88 OL lesions using a validated SMAD4 specific antibody. The staining was manifested as well-delineated nuclear staining. Based on the method described in the methods section, SMAD4 staining was graded as weak expression (Fig. 1A,) and strong expression (Fig. 1B). Forty-three (48.9%) of the OL lesions showed strong SMAD4 expression while other 45 (51.1%) OL lesions showed weak SMAD4 expression. SMAD4 expression was also measured in 22 OSCC samples (Fig. 1C) which were paired with their original OL samples. Paired t-test showed that the SMAD4 expression in OSCC samples was significantly lower than that in OL samples which went through malignant transformation (p<0.001). Among the 22 specimens of OL lesions, which later went through malignant transformation, 17 (77.3%) had strong SMAD4 expression, but only 26 (39.4%) of the 66 specimens of OL lesions, which did not go through malignant transformation during the follow-up period, had strong SMAD4 expression. The difference is statistically significant (Fig. 1D, p = 0.002).

### Evaluation of the factors in predicting OL malignant transformation risk

**Figure 2:**
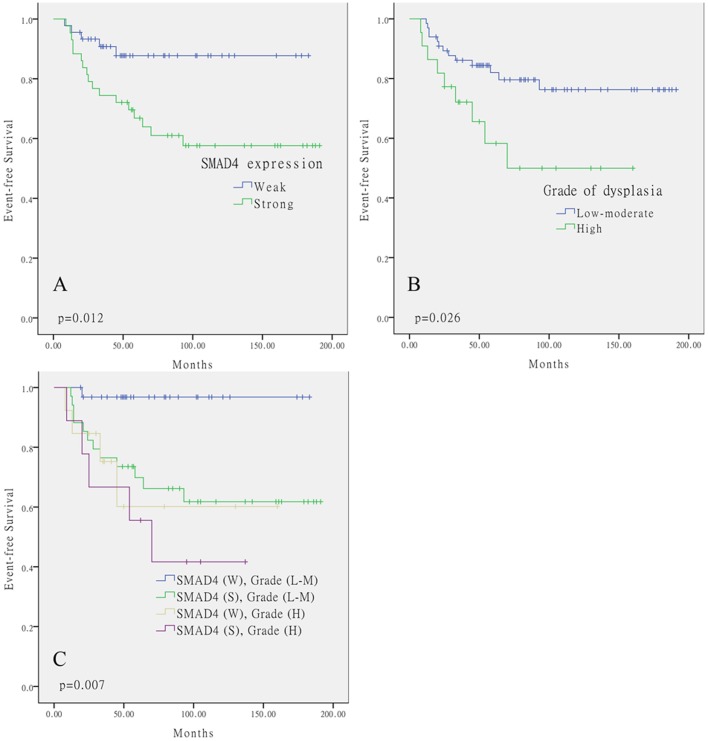
Kaplan–Meier analysis showed that A, Stronger expression of SMAD4 was associated with higher OL malignant transformed rate (p = 0.012, log-rank test); B, High-grade of dysplasia was associated with higher OL malignant transformed rate (p = 0.026, log-rank test). C, The combination of strong SMAD4 expression and high grade of dysplasia predicted the malignant transformation of oral leukoplakia better than either single factor did (p = 0.007, log-rank test).

To determine the predictor value of SMAD4 expression and clinicopathological characteristics in OL malignant transformation, EFS (Event-free survival) time was defined as the months from the time of OL diagnosis to the time OSCC diagnosis. Kaplan-Meier curves were performed to analyze for EFS using SMAD4 expression and clinicopathological characteristics. The current study showed that SMAD4 expression and the grade of dysplasia were significant predictors using log-rank test. Among the 88 OL specimens, 17 of 43 (39.5%) strong SMAD4 expression and 5 of 45 (11.1%) weak SMAD4 expression specimens went through malignant transformation (Fig.2A, p = 0.012). 9 of 22 (40.9%) high-grade OL lesions and 13 of 66 (19.7%) low and moderate-grade OL lesions went through malignant transformation (Fig.2B, p = 0.026).

Because of the grade of dysplasia was commonly used to assess the malignant transformation risk in OL lesions, it was then analyzed the two predictors simultaneously to determine whether SMAD4 expression pattern in OL specimens might augment the significance of OL risk prediction. We found that the combination of strong SMAD4 expression and high grade of dysplasia predicted the malignant transformation of oral leukoplakia better than either single factor did. (Fig.2C, p = 0.007).

In the univariate analysis, both SMAD4 expression (weak vs. strong) and the lesion histology (low and moderate-grade dysplasia vs. high-grade dysplasia) were significantly associated with OL malignant transformation, and SMAD4 expression was the most striking factor (Table 2, p = 0.018 and 0.032, respectively). Multivariate analysis indicated that both SMAD4 expression and grade of dysplasia were independent factors for predicting OL malignant transformation (p = 0.013 and 0.021, respectively) (Table 2).

**Table 2:** Cox proportional hazard regression models in analyzing OL malignant transformation risk.

Variables	p value	Hazard ratio	95% CI
**Univariate analysis**			
Age (≤55 v>55)	0.721	0.859	0.372–1.981
Sex (male v female)	0.061	0.406	0.158–1.044
Grade of dysplasia (L–M v H)*	**0.032**	0.394	0.168–0.923
Lesion site (tongue v nontongue)	0.514	0.753	0.322–1.764
Smoking (negative v positive)	0.199	2.603	0.605–11.199
Alcohol intake (negative v positive)	0.514	1.503	0.442–5.103
SMAD4 expression (weak v strong)	**0.018**	0.298	0.110–0.810
**Multivariate analysis**			
Grade of dysplasia (L–M v H)	**0.021**	0.367	0.157–0.861
SMAD4 expression (weak v strong)	**0.013**	0.282	0.104–0.769

CI: confidence interval.

* L–M: low and moderate-grade dysplasia, H: high-grade dysplasia.

Bold indicates values that are statistically significant (p<0.05).

## Discussion

TGF-beta signaling pathway plays an important role in embryonic development and in the regulation of tissue homeostasis[19], [20]. Previous reports showed that TGF-beta possessed dual functions: it functioned as a tumor suppressor in the initiation steps of tumorigenesis by inhibiting proliferation and inducing apoptosis while in the later stages of tumorigenesis and progression by inducting epithelium-mesenchymal transition (EMT), stimulating angiogenesis and suppressing immune system.

SMAD proteins are important mediators in TGF-beta signaling pathway and can be classified into different groups based on their roles in mediating TGF-beta superfamily components. SMAD2 and SMAD3 are classified as receptor activated SMADs (R-SMADs) because they are important substrates for the type I TGF-beta receptor while SMAD4 functions as common SMAD (Co-SMAD) to mediate TGF-beta and BMP signaling pathway. After receptor-induced phosphorylation, R-SMADs form complexes with the common-mediator SMAD4, which are translocated into nucleus, and regulate expression of target genes, such as beta-catenin[21], Id2[22] and keratin23[23] in cooperation with other transcription factors, co-activators and corepressors. SMAD4 was frequently inactivated in cancers and linked to activation of K-ras oncogene in pancreatic cancer[13]. In HNSCC, loss of heterozygosity at SMAD4 gene region was observed in 30%–50% of the tumors[17], [24], [25], suggesting a tumor suppressor role of SMAD4. However, SMAD4 gene mutation was uncommon in HNSCC[24], [26] in comparison with 35% point mutation rate in pancreatic cancer and 12% in colon cancer. In HNSCC, reduced SMAD4 expression was associated with more aggressive cancer phenotypes[27]. Recent studies showed that reduced SMAD4 mRNA levels were observed in 31/36 (86%) of the HNSCC samples and in 24/36 (67%) of the adjacent mucosa, and 61.12% OSCC exhibited SMAD4 loss[18], [28]. Our original hypothesis was that loss of SMAD4 expression might play a role in the malignant transformation in patients with OL and evaluation of SMAD4 expression might be useful in predicting the clinical outcome of OL lesions.

A surprising result in our study was the strong association between a higher SMAD4 expression and an increased rate of OL malignant transformation. We noted that most studies investigated established cancers and often found the loss of SMAD4 gene occurring at later stages of tumorigenesis[29], [30]. Few studies showed a loss of SMAD4 in premalignancies such as OLP lesions[31]. It is possible that in early oral tumorigenesis, carcinogen exposure triggers activation of TGF-beta signaling pathway and overexpression of SMAD4 in an attempt to inhibit tumorigenic process. Because OL is a clinical diagnosis, and the lesion is caused by various etiologic factors not always towards tumorigenesis, the up-regulation of SMAD4 we observed might be a carcinogen-induced process and therefore separating them from other lesions not in the carcinogen-induced process. However, the protection is insufficient to prevent OL malignant transformation because the damaged epithelial cells can acquire additional genetic and epigenetic alterations, leading to eventual OSCC development. To illustrate this possibility, we also detected SMAD4 expression in 22 OSCC samples which were paired with their original 22 OL samples. It showed that the SMAD4 expression in OSCC samples was significantly lower than that in their paired OL samples. The lower SMAD4 expression in OSCC tissues was in accordance with the other studies which showed that SMAD4 was a tumor suppressor in OSCC[18]. In addition, here for the first time we detected the SMAD4 expression in different stages of oral mucosal diseases within the same patient. We found that SMAD4 may function differently in earlier OL and later OSCC lesions. In earlier OL stage the higher SMAD4 reflected the protection reaction because of the dysplastic proliferation of the epithelial cells, while in the later OSCC stage the lower SMAD4 reported the dysfunction of the tumor suppress gene. It should be noted that many commercially available SMAD4 antibodies have limited specificity in immunohistochemistry assays, which will likely impact data interpretation. Before we started this study, we did test the specificity of SMAD4 isoforms from different companies using the tissue from SMAD4 knock-out mice[18] as a control and carefully selected rabbit polyclonal SMAD4 antibody from Santa Cruz (USA) to perform the immunostaining.

The use of Aperio ScanScope system and ImageScope software to quantify staining reduced potential bias from human reading and will make future use of such biomarker easier in regular pathology laboratories.

To the best of our knowledge, this study determined for the first time that SMAD4 expression could be used as a biomarker to predict malignant transformation risk in patients with OL, who received a long-term follow-up. Importantly, the prediction is independent of grade of dysplasia, which highlights the potential clinical value of the biomarker. The combination of strong SMAD4 expression and high grade of dysplasia was a better predictor for the malignant transformation of oral leukoplakia than either single factor. One limitation of this study was that we only focused on the significance between SMAD4 expression and the OL malignant transformation risk. However, SMAD4 was associated with other activated SMAD proteins, such as SMAD2 and SMAD3. Whether SMAD2 and SMAD3 are also correlated with the OL malignant transformation needs to be addressed in further study.

## Conclusions

In conclusion, besides the notion that SMAD4 was a tumor suppressor and loss of SMAD4 expression may lead to spontaneous oral squamous cell carcinoma development, patients whose oral leukoplakia lesions with higher levels of SMAD4 expression displayed a significantly higher rate of malignant transformation. Our results suggested that SMAD4 might be activated in early oral tumorigenesis but insufficient to halt carcinogenic process. The combination of SMAD4 expression and histological grade of dysplasia was a better predictor for the malignant transformation of oral leukoplakia.

## Patients and Methods

### Ethics statement

This study was approved by the Human Research Ethics Committee of Shanghai Ninth People's Hospital, Shanghai Jiao Tong University School of Medicine, and written informed consent was obtained from all patients.

### Patients and specimens

All 88 patients with clinical and pathological diagnosis of OL from the period of 1996 through 2010 were included in the present study. Clinicopathological characteristics and follow-up information were retrospectively reviewed in the Department of Oral Pathology, School of Stomatology, Shanghai Jiao Tong University School of Medicine, Shanghai, China. Clinicopathological parameters including age, sex, lesion site, history of smoking and alcohol intake and histological grade of dysplasia, were obtained from patients' medical charts and pathological reports. All the OL lesions were classified to two groups: low-moderate grade dysplasia and high grade dysplasia for analysis. All the patients with OL underwent biopsy or surgery, and the formalin-fixed paraffin-embedded tissue blocks were available.

### Immunohistochemistry

Formalin-fixed, paraffin-embedded tissues were cut into 4-µm tissue sections. After deparaffinization through a graded series of xylene, the sections were rehydrated in a graded series of alcohol. Heat mediated antigen retrieval using 0.01 M sodium citrate buffer (pH 6.0) was performed and endogenous peroxidase was quenched with 3% hydrogen peroxide for 20 minutes at room temperature. The sections were then incubated with 5% normal goat serum to reduce nonspecific binding. The sections were incubated with rabbit polyclonal SMAD4 antibody (1∶250, Santa Cruz, USA) at 4°C overnight, followed by biotinylated secondary antibody and then ABC reagent. Detection was accomplished with the avidin-biotin complex (ABC) system using Vectastatin elite ABC kit (Vector Laboratories). The expression of SMAD4 was detected with the diaminobenzidine chromogen system (Vector Laboratories). The sections were counterstained with haematoxylin, dehydrated and mounted. Blank controls were whole-tissue sections stained in the absence of the primary antibody.

### Quantitation of immunohistochemical staining

The slides were scanned using Aperio ScanScope CS Slide Scanner (Aperio Technologies, Vista, CA, USA). The ScanScope generated true color high resolution digital images of each stained sample, which were evaluated by Aperio ImageScope software (version 11.1.2.760). Nuclear v9 algorithm was used to calculate the percentage and intensity of nuclear-specific staining. The SMAD4 labeling index was defined as the weighted percentage of epithelium cells displaying nuclear staining multiplied by the degree of the staining intensity. For the mean SMAD4 labeling index of all those samples was 40, the SMAD4 expression was classified as weak expression (labeling index ≤40) and strong expression (labeling index >40).

### Statistical analysis

Statistical analysis was performed with SPSS 17.0 (SPSS Inc., Chicago, IL). The chi-square test was used to determine the relationship between the expression of SMAD4 and clinicopathological parameters. Event-free survival (EFS, or malignant transformation-free survival) was determined as the outcome variable. The association between SMAD4 expression and EFS were estimated using the log-rank test. All the variables were subjected to a multivariate analysis using the Cox proportional hazards regression model. The Enter method was used to determine a final Cox model. The HRs with their corresponding 95% CIs and *P* values were reported. All of the tests were two sides and a p-value of <0.05 was considered statistically significant.

## Supporting Information

Table S1 **Detailed clinicopathological characteristics and follow-up information for each patient.**
(XLS)
